# Diagnostic implication of fibrin degradation products and D-dimer in aortic dissection

**DOI:** 10.1038/srep43957

**Published:** 2017-03-06

**Authors:** Jian Dong, Xianli Duan, Rui Feng, Zhiqing Zhao, Xiang Feng, Qingsheng Lu, Qing Jing, Jian Zhou, Junmin Bao, Zaiping Jing

**Affiliations:** 1 Department of Vascular Surgery, Changhai Hospital, Second Military Medical University, Shanghai 200433, China; 2 Department of Surgery, Changhai Hospital, Second Military Medical University, Shanghai 200433, China; 3 Department of Cardiology, Changhai Hospital, Second Military Medical University, Shanghai 200433, China; 4 Key Laboratory of Stem Cell Biology and Laboratory of Nucleic Acid and Molecular Medicine, Institute of Health Sciences, Shanghai Institutes for Biological Sciences, Chinese Academy of Sciences & Shanghai Jiao-Tong University School of Medicine, Shanghai 200092, China

## Abstract

Fibrin degradation products (FDP) and D-dimer have been considered to be involved in many vascular diseases. In this study we aimed to explore the diagnostic implication of FDP and D-dimer in aortic dissection patients. 202 aortic dissection patients were collected as the case group, 150 patients with other cardiovascular diseases, including myocardial infarction (MI, n = 45), pulmonary infarction (n = 51) and abdominal aortic aneurysm (n = 54) were collected as non-dissection group, and 27 healthy people were in the blank control group. The FDP and D-dimer levels were detected with immune nephelometry. Logist regression analysis was performed to evaluate the influence of FDP and D-dimer for the aortic dissection patients. ROC curve was used to determine the diagnostic value of FDP and D-dimer. The FDP and D-dimer levels were significantly higher in aortic dissection patients than in non-dissection patients and the healthy controls. FDP and D-dimer were both the risk factors for patients with aortic dissection. From the ROC analysis, diagnostic value of FDP and D-dimer were not high to distinguish aortic dissection patients from the non-dissection patients. However FDP and D-dimer could be valuable diagnostic marker to differentiate aortic dissection patients and healthy controls with both AUC 0.863.

Aortic dissection is one of the most complex and dangerous cardiovascular diseases. The incidence and mortality are both very high, although it has well-established treatment guidelines^1^,^2^,^3^,^4^,^5^. It is reported that if the aortic dissection patients do not have early diagnosis or appropriate management, it is rapidly fatal^6^,^7^. But about 20% patients are without pain and with non-evocative symptoms, like syncope, cerebrovascular accidents, and congestive heart failure. Thus to evaluate the patients with suspicion of aortic dissection is very difficult^6^,^8^. Aortic dissection usually is divided into 2 types with the Stanford classification, Stanford type A and B. Type A has high mortality, but life-saving surgical repair is allowed with the rapid diagnosis^9^. Type B with a long-term prognosis is correlated with both higher morbidity and mortality^10^,^11^,^12^,^13^.

Thus with the high morbidity and mortality, early diagnosis is very important for improve the treatment and survival of aortic dissection patients^14^. D-dimer is a specific protein fiber degradation product of cross linked fibrin by the hydrolysis of fibrinolytic enzyme^15^,^16^. When thrombus degrade, D-dimer can release into the circulatory system^17^. In normal blood, the level of D-dimer is low, but once the thrombosis occurs, the D-dimer level is increased^18^. Studies have found that in the patients with cardiovascular and cerebrovascular diseases, D-dimer level is increased. And the increased D-dimer level has direct association with prognosis.

Recent years, biochemical diagnosis is widely used in the early diagnosis of many diseases, fibrin degradation products (FDP) is the degradation products of fibrous protein. Many reports have showed that FDP is involved in many vascular diseases. FDP is a mitogen of many cell types, and can promote the proliferation of endothelial cells, smooth muscle cell, and fibroblast, and cholesterol deposition^19^. FDP also can induce the adhesion and gather of leucocytes, which results in damage of blood vessel endothelium^20^.

However it is fewer reports of the both FDP and D-dimer and aortic dissection. Thus in this study, we described our preliminary experience with the diagnostic implication of FDP and D-dimer for aortic dissection.

## Methods

### Study Population

This was a restrospective study. The following methods were carried out in accordance with the approved guidelines. This study was approved by the Ethics Committee of the Changhai Hospital, written informed consent was obtaining from every subject.

This study recruited a total of 202 patients who were diagnosed with aortic dissection in our hospital from 2007–2016 as the case group with the age of 55.32 ± 13.59 (146 males and 56 females). And 150 patients diagnosed with other cardiovascular diseases including myocardial infarction (MI, n = 45), pulmonary infarction (n = 51) and abdominal aortic aneurysm (n = 54) were collected as non-dissection group with the age of 64.33 ± 14.31 (114 males and 36 females). There were 27 healthy volunteers as the control group with the age of 64.86 ± 10.52 (21 males and 6 females).

Aortic dissection patients were diagnosed by pathologists according to standard guidelines assisting with physical examination and contrast-enhanced computed tomography scan. The diagnosis of other cardiovascular diseases, like MI, pulmonary infraction and abdominal aortic aneurysm were performed using specific biomarkers, echocardiography, coronary angiography, CT or MRI according to respective guidelines.

### Clinical studies in patients with aortic dissection

In this study, we collected the clinical data of the aortic dissection patients, and the data was all listed in Table 1. The following data obtained: age, HDL, LDL, PT, TT, FIB, INR, cholesterol and triglyceride. In the case group, there were 3 types aortic dissection patients, including patients without complications, with recurrence or endoleak, and dead patients.

### Measurements and analysis of FDP and D-dimer

The blood samples were collected from the patients with aortic dissection, non-dissection group and the healthy group. Immune nephelometry was performed in this detection. The FDP level and D-dimer level of all the volunteers were measured with STA R-automatic coagulometer (STAGO, Germany) using the FDP kit and D-D kit (Sekisui Medical Co. LTD).

### Statistical analysis

All statistical analyses were performed using the software of SPSS 19.0 and GraphPad Prism 5. The data are summarized and presented as means ± SD. In order to compare the FDP and D-dimer level between the case group and non-dissection group and healthy group, respectively, one-way ANOVA was used. The clinicopathological characteristics among the three groups were assessed using Chi-square tests. Logist regression analysis was performed to evaluate the influence of FDP and D-dimer for the aortic dissection patients. Receiver operating characteristic (ROC) analysis was performed to determine the diagnostic value of FDP and D-dimer in distinguishing aortic dissection patients and non-dissection group and healthy group. In this study all *P* values < 0.05 were considered statistically significant.

## Results

### Clinical data of patients in the three groups

In our study, the clinical data of the three groups were listed in Table 1. And they were assessed using Chi-square tests. From the results, we could see that among the clinical data, age, PT and FIB had significantly differences among the case group, non-dissection group and healthy group. Other clinical data, including HDL, LDL, TT, INR, cholesterol and triglyceride, all had no significantly differences among the three groups.

### FDP and D-dimer levels in the three groups

**Figure 1:**
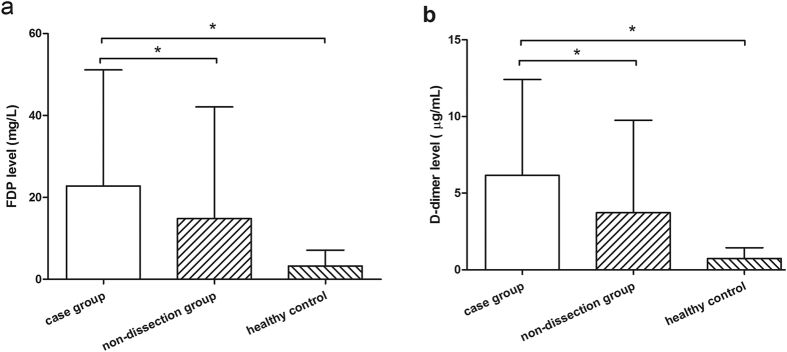
The FDP and D-Dimer levels in the three group, the case group, non-dissection group, and the healthy group. (**a**) FDP levels; (**b**) D-Dimer levels.

The FDP and D-dimer levels of the three groups were showed in the Fig. 1. The results of Fig. 1a revealed that the FDP level of the patients with aortic dissection (22.81 ± 28.31 mg/L) was significantly higher than that both in non-dissection group (14.81 ± 27.26 mg/L) and the healthy group (3.21 ± 3.91 mg/L) (*P* < 0.05), although the FDP level was also higher in non-dissection group than that of healthy group. And in Fig. 1b, the D-dimer had the same results with FDP. The D-dimer level of the patients with aortic dissection (6.16 ± 6.24 g/mL) was also significantly higher than that both in non-dissection group (3.72 ± 6.03 g/mL) and the healthy group (0.73 ± 0.70 g/mL) (*P* < 0.05).

### The logistic regression of FDP and D-dimer in aortic dissection patients

In the case group, the OR value of FDP (*P* = 0.011) and D-dimer (*P* = 0.006) were 1.197 and 2.040, respectively, 95%CI were 1.042–1.374 and 1.223–3.401, respectively. And in the non-dissection group, the FDP and D-dimer also were risk factors as similar with those in the case group. The results suggested that FD and D-dimer were both risk factors not only in the non-dissection group but also in the case-group according to the analysis of logist regression analysis (Table 2).

### Diagnostic performance of FDP and D-dimer

**Figure 2:**
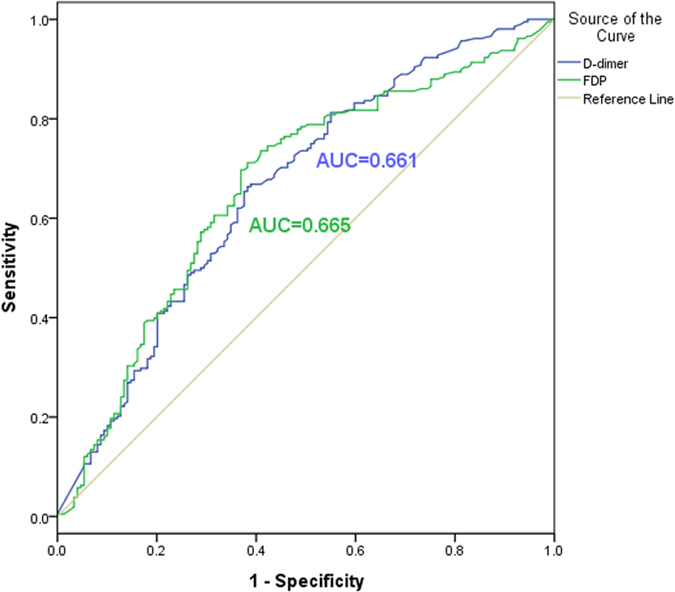
The ROC curves of FDP and D-Dimer to distinguish aortic dissection patients and non-dissection group.

**Figure 3:**
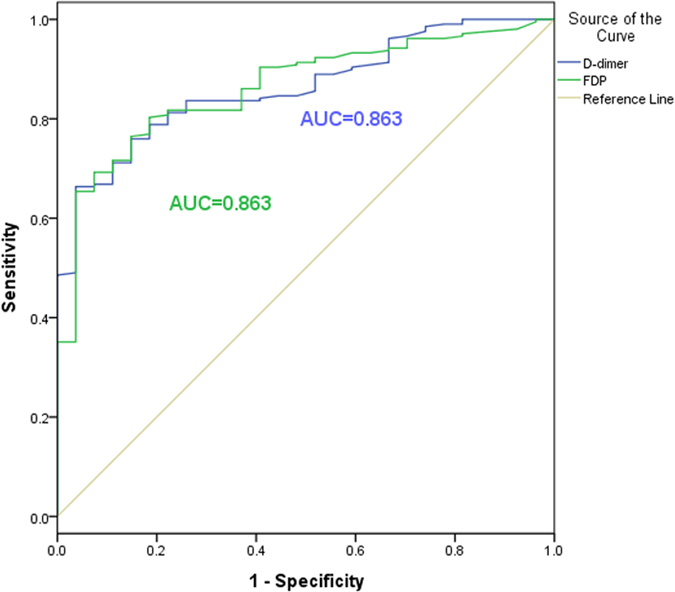
The ROC curves of FDP and D-Dimer to distinguish aortic dissection patients and healthy group.

In order to estimate the diagnostic implication of FDP and D-dimer in aortic dissection patients, ROC analysis was performed (Figs 2 and 3). Figure 2 showed the ROC of FDP and D-dimer between case group and non-dissection group with the area under the curve (AUC) of 0.661 and 0.665, respectively. And in the Fig. 3, the results revealed the ROC of FDP and D-dimer between case group and healthy group with the both AUC of 0.863. For FDP, in the analysis of case group and non-dissection group, the sensitive and specificity were 53.1 and 73.6%, and in the analysis of case group and healthy group were 92.6 and 69.2%, respectively. For D-dimer, the the sensitive and specificity of case group and non-dissection group were 68.8 and 60.9%, and 68.3 and 96.4% in case group and healthy group.

## Discussion

Aortic dissection is still a potentially catastrophic cardiovascular disease^21^,^22^,^23^. According to the foreign data, the incidence of aortic dissection can be 100–290/1000 thousand people^2^, and in America there are 10 thousand new case of aortic dissection every year^24^. Based on the statistics, the incidence of males is higher than that of females, about 2–3:1, and the patients with type A are more than those with type B^25^. Because of the complex clinical features, the rates of missed diagnosis and misdiagnosis are high, and the mortality of untreated acute aortic dissection is 21% per day, 37% of two days and 74% of a week^26^. Although diagnosis and treatment of aortic dissection have greatly improved in recent years, it is still difficult to recognize at clinical presentation for aortic dissection^21^,^27^,^28^,^29^.

FDP has been reported to be involved in vascular diseases. Fukujima *et al*. found that FDP participate the development and progression of atherosclerosis and thrombus^30^. Corban MT *et al*. also found that FDP was associated with larger coronary plaques and greater plaque necrotic core^31^. In addition, in the study of Akiyoshi Hagiwara *et al*., they showed that FDP was a valuable diagnosis biomarker for patent-type acute aortic dissection patients and thrombosed-type acute aortic dissection patients. They also found a strong correlation between FDP and D-dimer in acute aortic patients^32^. D-dimer is a kind of fibrin degradation product. In the 1990 s, it was first introduced to be a diagnostic aid for diseases such as pulmonary embolus and deep venous thrombosis^33^,^34^. Studies have found that increased D-dimer level was revealed in many diseases, including cancers, deep venous thrombosis, disseminated intravascular coagulation, aortic dissection, and so on. Weber *et al*. found that the increased D-dimer level had association with aortic dissection^35^. In the research of Eggebrecht *et al*., D-dimer level was elevated in all patients with aortic dissection^36^.

In this study, we aimed to explore the diagnostic implication of FDP and D-dimer for the aortic dissection patients. FDP and D-dimer levels were both obviously higher in aortic dissection patients than the patients with myocardial infarction, pulmonary infarction and abdominal aortic aneurysm as well as the healthy people. And among the clinical data, age, PT and FIB had significantly differences among the three groups. The results indicated FDP and D-dimer were both the risk factors for the patients with aortic dissection. From the ROC, in the analysis of case group and non-dissection group, the diagnostic value of FDP and D-dimer were not very high for distinguishing aortic dissection patients and non-dissection group. However, in the analysis of case group and healthy group, the specificity of D-dimer was 96.4%, and the sensitive of FDP was 92.6%, and the AUC of both FDP and D-dimer were 0.863. Therefore, the diagnostic value of FDP and D-dimer were outstanding to distinguishing aortic dissection patients and healthy people. Our findings were compatible with the previous studies and in order to improve the sensitive and specificity, the combined detection of FDP and D-dimer should be applied to clinic in the follow research.

Although in this study, it was revealed that FDP and D-dimer had diagnostic implication for aortic dissection patients, more and further studies for aortic dissection should be performed in the future.

## Additional Information

**How to cite this article:** Dong, J. *et al*. Diagnostic implication of fibrin degradation products and D-dimer in aortic dissection. *Sci. Rep.* **7**, 43957; doi: 10.1038/srep43957 (2017).

**Publisher's note:** Springer Nature remains neutral with regard to jurisdictional claims in published maps and institutional affiliations.

## Figures and Tables

**Table 1:** Comparison of clinical data among the three group volunteers.

Factor	Group			*P* value
	Case group	Non-dissection group	Healthy group	
age	55.32 ± 13.59	64.33 ± 14.31	64.86 ± 10.52	0.002*
HDL	1.20 ± 0.52	1.12 ± 0.35	1.24 ± 0.42	0.906
LDL	2.49 ± 0.77	2.62 ± 0.89	2.52 ± 0.81	0.665
PT	14.65 ± 5.59	14.35 ± 2.95	13.09 ± 2.65	0.023*
TT	18.21 ± 12.12	16.99 ± 3.05	15.84 ± 1.12	1.000
FIB	4.18 ± 3.88	4.09 ± 1.49	4.88 ± 3.42	0.045*
INR	1.17 ± 0.83	1.13 ± 0.32	1.06 ± 0.21	0.228
Cholesterol (mmol/L)	4.26 ± 1.07	4.43 ± 1.19	4.98 ± 1.21	0.390
Triglyceride (mmol/L)	1.32 ± 0.74	1.36 ± 0.85	1.59 ± 1.10	0.123

**Table 2:** Logist regression analysis of the FDP and D-Dimer.

	OR	95%CI	*P* value
Case group			
FDP	1.197	1.042–1.374	0.011
D-Dimer	2.040	1.223–3.401	0.006
Non-dissection group			
FDP	1.186	1.032–1.361	0.016
D-Dimer	1.888	1.133–3.418	0.015

OR: odds ratio.
